# Interventions to decrease the risk of adverse cardiac events for patients receiving chemotherapy and serotonin (5-HT3) receptor antagonists: a systematic review

**DOI:** 10.1186/2050-6511-16-1

**Published:** 2015-01-26

**Authors:** Andrea C Tricco, Charlene Soobiah, Wing Hui, Jesmin Antony, Vladi Struchkov, Brian Hutton, Brenda Hemmelgarn, David Moher, Sharon E Straus

**Affiliations:** Li Ka Shing Knowledge Institute, St. Michael’s Hospital, 209 Victoria Street, East Building, Toronto, Ontario M5B 1 W8 Canada; Clinical Epidemiology Program, Centre for Practice-Changing Research, Ottawa Hospital Research Institute, 725 Parkdale Ave., Ottawa, Ontario K1Y 4E9 Canada; Departments of Medicine and Community Health Sciences, University of Calgary, TRW Building, 3rd Floor, 3280 Hospital Drive NW, Calgary, Alberta T2N 4Z6 Canada; Department of Geriatric Medicine, Faculty of Medicine, University of Toronto, 27 Kings College Circle, Toronto, Ontario M5S 1A1 Canada

**Keywords:** Serotonin receptor antagonists, Chemotherapy, Adverse events, Cardiac harm, Systematic review, Electrocardiogram

## Abstract

**Background:**

Patients may experience nausea and vomiting when undergoing chemotherapy or surgery requiring anesthesia. Serotonin 5-hydroxytryptamine 3 (5-HT3) receptor antagonists are effective antiemetics, yet may cause adverse cardiac events, such as arrhythmia. We aimed to identify interventions that mitigate the cardiac risk of 5-HT3 receptor antagonists.

**Methods:**

Electronic databases, trial registries, and references were searched. Studies on patients undergoing chemotherapy or surgery examining interventions to monitor cardiac risk of 5-HT3 receptor antagonists were included. Search results were screened and data from relevant studies were abstracted in duplicate. Risk of bias of included studies was assessed using the Cochrane Effective Practice and Organisation of Care (EPOC) group’s risk-of-bias tool. Due to a dearth of included studies, meta-analysis was not conducted.

**Results:**

Two randomized clinical trials (RCT) and 1 non-randomized clinical trial (NRCT) were included after screening 7,637 titles and abstracts and 1,554 full-text articles. Intravenous administration of different dolasetron doses was examined in the NRCT, while dolasetron versus ondansetron and palonosetron versus ondansetron were examined in the RCT. Electrocardiogram (ECG) was the only intervention examined to mitigate cardiac harm. No differences in ECG evaluations were observed between dolasetron or palonosetron versus ondansetron after 15 minutes, 24 hours, and 1 week post-administration in the 2 RCTs. Four deaths were observed in one RCT, which were deemed unrelated to palonosetron or ondansetron administration. Minor increases in PR and QT intervals were observed in the NRCT for dolasetron dosages greater than 1.2 mg/kg 1–2 hours post-administration, but were deemed not clinically relevant.

**Conclusions:**

ECG monitoring of chemotherapy patients administered with 5-HT3 receptor antagonists did not reveal clinically significant differences in arrhythmia between the medications at the examined time periods. The usefulness of ECG to monitor chemotherapy patients administered with 5-HT3 receptor antagonists remains unclear, as all patients received ECG monitoring.

**Trial registration:**

PROSPERO registry number: CRD42013003565

**Electronic supplementary material:**

The online version of this article (doi:10.1186/2050-6511-16-1) contains supplementary material, which is available to authorized users.

## Background

Nausea and vomiting are common adverse effects following chemotherapy or surgery requiring anesthesia [1, 2]. Serotonin 5-hydroxytryptamine 3 (5-HT3) receptor antagonists can effectively prevent nausea and vomiting for patients undergoing these interventions [1, 3–5]. However, a prolonged QT interval has been observed in previous studies of these medications [6, 7]. The results of these studies suggest that patients who are administered 5-HT3 receptor antagonists might be at risk of experiencing cardiac harm, yet this has not been confirmed in large-scale studies or systematic reviews.

Diagnostic tests can be used to monitor or mitigate cardiac risk that might be associated with 5-HT3 receptor antagonist administration. For example, electrocardiogram (ECG) provides information on PR and QT prolongation, which may lead to arrhythmia (e.g., torsade de pointes tachycardia) [8], all-cause mortality [9], and sudden death [10]. Cardiac telemetry monitors provide continuous ECG monitoring for 24 hours or longer [11]. Electrolyte imbalances, such as hypocalcemia, hypomagnesemia and hypokalemia can result from persistent vomiting and these abnormalities can cause QT interval prolongation [12]. As such, monitoring might be necessary in patients receiving 5-HT3 antagonists and who are vomiting. However, these diagnostic tests are burdensome to the healthcare system and patients. We aimed to determine whether diagnostic interventions can be implemented to mitigate the risk of adverse cardiac events associated with 5-HT3 receptor antagonists for patients undergoing chemotherapy or surgery through a systematic review.

## Methods

We used the Preferred Reporting Items for Systematic Reviews and Meta-analysis (PRISMA) Statement to report the results of our systematic review [13].

### Protocol

We developed a protocol that was reviewed by clinicians, systematic review methodologists, pharmacoepidemiologists, and Health Canada. We registered our protocol with the PROSPERO database (CRD42013003565) and published the final version [14].

### Eligibility criteria

Studies were included if they examined interventions to monitor cardiac risk associated with 5-HT3 receptor antagonists, such as ECG monitoring, telemetry, adjustment of antiarrhythmics, and electrolyte monitoring and replacement. Studies including patients of all ages receiving 5-HT3 antagonist receptors for nausea and vomiting symptoms post-surgery or after chemotherapy and reporting on arrhythmia (primary outcome), sudden cardiac death, QT prolongation, PR prolongation, and/or all-cause mortality were eligible for inclusion. Experimental studies (randomized clinical trials, quasi-randomized clinical trials, non-randomized clinical trials), quasi-experimental studies (interrupted time series, controlled before and after studies), and cohort studies were included regardless of whether they were unpublished or written in languages other than English.

### Information sources and literature search

MEDLINE, EMBASE, and Cochrane Central were searched from inception onwards. The electronic literature search was supplemented by searching trial registers and scanning the reference lists of included studies.

An experienced librarian drafted the search strategies, which were peer reviewed by another expert librarian using the Peer Review of Electronic Search Strategies (PRESS) checklist [15]. The final literature search for MEDLINE has been published previously [14] and is available in the Additional file 1. Literature searches for the other databases can be obtained from the corrsponding author upon request.

### Study selection process

The eligibility criteria were calibrated on a random sample of 50 titles and abstracts from the literature search. They were revised to enhance clarity and readability by the team. Subsequently, each title and abstract was screened by two team members, independently. Conflicts were resolved by team discussion. The same process was followed for screening the potentially relevant full-text articles.

### Data items and data collection process

Following a similar process to screening, two team members independently abstracted data on study characteristics (e.g., setting, country where the study was conducted, details on the 5-HT3 medications, comparator used, and type of test conducted to assess cardiac risk), patient characteristics (e.g., mean/median age, percent female, type of surgery, type of cancer), and outcome results (e.g., number of patients experiencing arrhythmia, mean and standard deviation for PR prolongation). Companion reports (i.e., duplicate publications reporting the data on the same group of patients) were sorted and authors were contacted for data clarifications.

### Methodological quality/risk of bias appraisal

Studies were appraised using the Cochrane Effective Practice and Organisation of Care Risk of Bias Tool [16] and the McHarm tool for reporting adverse events [17].

### Synthesis of included studies

We were unable to conduct meta-analysis because none of the studies examined interventions to mitigate harm at the same time point. Furthermore, none of the studies compared an intervention to mitigate harm with a control group. As such, the studies were synthesized descriptively, with a focus on the study characteristics, patient characteristics, and outcome results.

## Results

### Literature search

**Figure 1:**
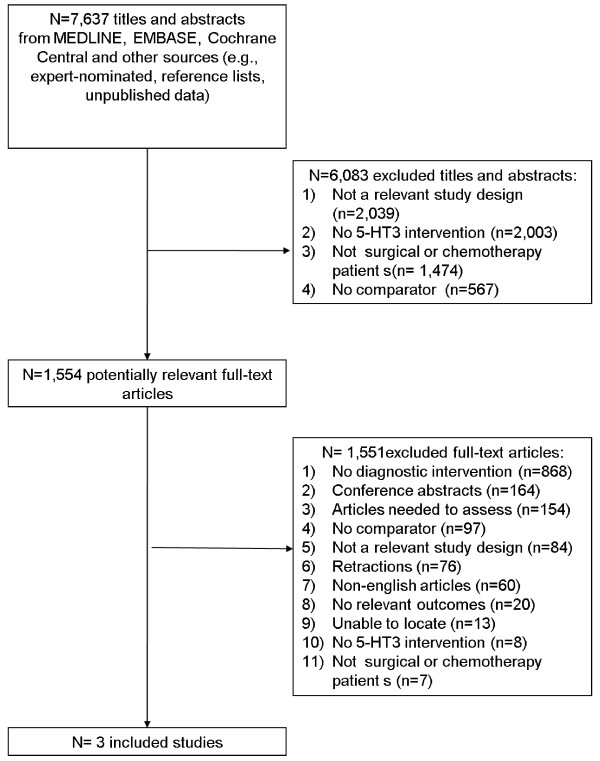
**Study flow.**

Two randomized clinical trials and 1 non-randomized clinical trial [18–20] were included after screening 7,637 titles and abstracts and 1,554 full-text articles (Figure 1).

### Study and patient characteristics

The study conduct period was 2000 and 2002 in the randomized clinical trials [19, 20] and not reported in the non-randomized clinical trial [18] that was published in 1996 (Table 1). The trials were conducted in the United States [18], multiple European countries [19], and South Korea [20]. One trial examined intravenous dolasetron at 1.2, 1.8, and 2.4 mg/kg administered 30 minutes prior to chemotherapy [18] and another examined intravenous palonosetron 0.25 to 0.75 mg compared to ondansetron 32 mg administered 30 minutes prior to chemotherapy [19]. The third trial examined the effects of intravenous dolasetron 100 mg administered 30 minutes prior to chemotherapy and up to 200 mg orally 2 to 5 days post-chemotherapy compared with another group who received 8 mg of intravenous ondansetron 30 minutes prior to chemotherapy and up to 16 mg intravenously 2–4 hours post-chemotherapy plus an additional 16 mg orally 2–5 days after chemotherapy.

**Table 1:** **Study characteristics**

Reference	Study period, country	Study design, # of patients	5-HT3 dose/day	Intervention, examination timing	Outcomes examined
Hesketh [18]	NR, USA	Non-RCT, 44	IV dolasetron 1.2 mg/kg, 1.8 mg/kg, 2.4 mg/kg 30 mins before chemotherapy	ECG, 1–2 hrs and 24–48 hrs	PR, QT
Gralla [19]	August 2000 to October 2001, Germany, Italy, UK, Netherlands, Russia	RCT, 98	IV palonosetron 0.25 mg, IV palonosetron 0.75 mg, IV ondansetron 32 mg 30 mins before chemotherapy	ECG, 15 mins, 24 hrs, 1 wk	Mortality, QT
Kim [20]	April 2002 to October 2002, South Korea	RCT, 114	IV dolasetron 100 mg 30 mins before and 200 mg p.o. 2-5 days after chemotherapy, IV ondansetron 8 mg 30 mins before and IV ondansetron 16 mg 2–4 hrs plus an additional 16 mg/day p.o. 2-5 days after chemotherapy	ECG, 15 mins, 24 hrs, 1 wk	ECG findings unspecified

ECG: electrocardiogram; IV: intravenous; NR: not reported; Non-RCT: non-randomized clinical trial; p.o.: administered orally; RCT: randomized controlled trial.

The only intervention identified to mitigate cardiac risk that was examined in the trials was an ECG at various time points. ECG monitoring was not compared with placebo, usual care or another type of diagnostic intervention. The percentage of females included in the trials ranged from 37 to 72% (Table 2). All trials included adult cancer patients with a median (or mean) age ranging from 54 to 56 years. All were receiving a 5-HT3 receptor antagonist for their chemotherapy treatment for various cancer sites. Patients were receiving a variety of chemotherapeutic agents.

**Table 2:** **Patient characteristics**

Reference	% female	Mean age in years (SD) [range]	Cancer site (%)	Common chemotherapeutic agents* (%)
Hesketh [18]	71	Median 54 (NR) [25–81]	Breast (55), other (45)	Doxorubicin (15), cyclophosphamide (26), 5-FU (43), vincristine (23), methotrexate (14)
Gralla [19]	72	55 (11) [18-NR]	Breast (57), lung (80 < bladder (5), colon (4), rectal (3), small-cell lung (3), gastric (3)	Cyclophosphamide (63), doxorubicin (48), cisplatin (18), methotrexate (16), carboplatin (12)
Kim [20]	37	55.6 (11) [18-NR]	Lung (26), gastric (11), other (29)	5-FU plus cisplatin (27), cisplatin plus the following: taxol, docetaxel, etoposide and ifosfomide, etoposide, gemcitabine, docetaxel plus 5-FU (73)

NR: not reported; SD: standard deviation. *received by >10% of patients.

### Risk of bias assessment

All of the included trials received the same intervention (ECG monitoring) without a comparator group and were assessed for risk of bias (Table 3). The non-randomized clinical trial had the highest risk of bias, with four items assessed as having a high risk of bias [18]. One of the randomized trials had a high risk of bias for allocation concealment and blinding and an unclear risk of bias for random sequence generation and contamination [20]. The other randomized trial had an unclear risk of bias for random sequence generation, contamination, and selective outcome reporting [19]. All of the other risk of bias criteria was assessed as unclear.

**Table 3:** **Appraisal of risk of bias, according to Cochrane Effective Practice and Organization of Care risk-of-bias tool** [16]

Criterion	Hesketh [18]	Gralla [19]	Kim [20]
Random sequence generation	High	Unclear	Unclear
Allocation concealment	High	Low	High
Similar baseline outcome measures	Unclear	Low	Low
Similar baseline characteristics	Low	Low	Low
Incomplete outcome data	Low	Low	Low
Blinding	High	Low	High
Contamination	Unclear	Unclear	Unclear
Selective outcome reporting	Unclear	Unclear	Unclear
Other bias	High	Low	Low

### Arrhythmia

None of the included trials reported data on arrhythmia, our primary outcome of interest. However, all three trials reported no clinically relevant differences in ECG findings among the patients receiving the 5-HT3 receptor antagonists examined.

### Sudden cardiac death and all-cause mortality

Mortality was reported in one of the trials [19]. One withdrawal due to a serious adverse event (pulmonary embolism resulting in death) occurred in the ondansetron group and three other deaths occurred, yet the number of deaths per treatment group was not reported. All deaths were deemed as unlikely or definitely unrelated to the study medications [19]. Although mortality was not specifically reported, no patients dropped out due to mortality in the other two trials [18, 20].

### QT prolongation

One trial reported no differences between treatment groups in the mean post-dose change from baseline in the QT interval, which was adjusted using the Fridericia correction [19]. The values were 1 ms for palonosetron 0.25 mg, 2 ms for palonosetron 0.75 mg, and 5 ms for ondansetron. Another trial reported median increases in the corrected QT interval as being 11 ms for dolasetron 1.2 mg/kg, 27 ms for dolasetron 1.8 mg/kg, and 39 ms for dolasetron 2.4 mg/kg [18]. These were considered asymptomatic and not clinically significant. The third trial did not specifically report on this outcome but reported no relevant differences between the treatment groups with respect to ECG findings [20] (Table 4).

**Table 4:** **QT and PR prolongation**

Author, year	Treatment (dose, time of treatment)		
**Hesketh** [18]			
	Dolasetron (1.2 mg/kg, BC)	Dolasetron (1.8 mg/kg, BC)	Dolasetron (2.4 mg/kg, BC)
QT prolongation*	11 ms	27 ms	39 ms
PR prolongation^†^	1 ms	10 ms	22 ms
**Gralla** [19]			
	Palonosetron (0.25 mg, BC)	Palonosetron (0.75 mg, BC)	Ondansetron (32 mg, BC)
QT prolongation*	1 ms	2 ms	5 ms
PR prolongation	NS	NS	NS
**Kim** [20]			
	Dolasetron (100, BC+ 800 mg, AC)	Ondansetron (8 mg, BC + 80 mg, AC)	
QT prolongation	NS	NS	
PR prolongation	NS	NS	

AC: after chemotherapy treatment; BC: before the start of chemotherapy treatment; NS: not significant. *mean change from baseline; ^†^median change from baseline.

### PR prolongation

One trial reported median increases in the PR interval as being 1 ms for dolasetron 1.2 mg/kg, 10 ms for dolasetron 1.8 mg/kg, and 22 ms for dolasetron 2.4 mg/kg [18]. A significant dose–response relationship was found between dolasetron and the PR interval at 1–2 hours post-administration. These changes were considered asymptomatic and not clinically significant. The other trials did not specifically report on this outcome but reported no relevant differences between the treatment groups with respect to ECG findings [19, 20] (Table 4).

## Discussion

In our systematic review, we did not find any clinically relevant changes in the QT interval between different dosages of dolasetron [18] or between palonosetron and ondansetron [19] or dolasetron and ondansetron [20]. Furthermore, we did not find any other significant differences in other ECG changes between these 5-HT3 receptor antagonists at 15 minutes, 1–2 hours, 24 hours, and 1 week post-administration. However, this does not suggest that these agents do not cause cardiac harm. For example, two case series have noted a prolonged QT interval after the administration of 5-HT3 receptor antagonists [6, 7]. Both articles were excluded here because they were case series and therefore there was not a comparison group and they did not examine interventions to mitigate the cardiac risk associated with these agents.

We had originally hoped to identify studies in which all patients received a 5-HT3 receptor antagonist and compared an intervention to mitigate cardiac harm (e.g., ECG monitoring) with placebo, usual care or another diagnostic intervention. As we did not identify any such study, the utility of the ECG to monitor adult chemotherapy patients after being administered a 5-HT3 receptor antagonist is currently unclear. Further research is required to recommend ECG monitoring for all adult chemotherapy patients administered a 5-HT3 receptor antagonist, as this would be burdensome to the healthcare system and to patients.

We identified many gaps in the literature in this area. For example, we did not identify studies that tested monitoring cardiac function after the administration of these agents other than the ECG. Similarly, we did not identify studies conducted among children receiving chemotherapy or patients of any age undergoing surgery requiring anesthesia and none of the included studies reported the proportion of patients with prolonged QT or PR intervals. Although unpublished studies were sought, none were identified that examined this research question.

As expected, the non-randomized clinical trial had the greatest risk of bias [18], as random sequence generation, allocation concealment, and blinding was not conducted. The two randomized clinical trials would be improved by adequately reporting random sequence generation, reporting whether contamination was a factor, and providing a protocol so that the potential for outcome reporting bias could be assessed [19, 20]. Limitations of our systematic review process include the few studies identified for inclusion. As well, our objective was to compare interventions to mitigate cardiac risk across intervention and comparator groups versus examine cardiac risk after the administration of these agents. As such, we excluded case series, case reports, and cross-sectional studies. We were unable to conduct meta-analysis or assess for publication bias because of the few studies included here.

## Conclusions

The utility of the ECG monitoring to mitigate cardiac harm among chemotherapy patients after being administered a 5-HT3 receptor antagonist is unclear; few studies exist that met our eligibility criteria. Future research is necessary to determine whether ECG (or other diagnostic interventions) are beneficial and whether these interventions should be used to monitor other patient populations, including children undergoing chemotherapy and patients of all ages receiving surgery requiring anesthesia.

## Electronic supplementary material

Additional file 1: **Literature search for MEDLINE.** (DOCX 16 KB)
