# Supplementary material for: Interventions to decrease the risk of adverse cardiac events for patients receiving chemotherapy and serotonin (5-HT3) receptor antagonists: a systematic review
Source: BMC Pharmacol Toxicol. 2015 Jan 26;16:1. doi: 10.1186/2050-6511-16-1 (PMC4417335; doi:10.1186/2050-6511-16-1)
Supplement: Supplementary file 1 — Additional file 1: Literature search for MEDLINE. (DOCX 16 KB) [file 40360_2014_366_MOESM1_ESM.docx]

**Appendix: Literature search for MEDLINE**

1 Ondansetron/

2 ondansetron.mp.

3 zofran.mp.

4 SN-307.mp.

5 SN307.mp.

6 GR38032F.mp.

7 GR-38032F.mp.

8 GR C50775.mp.

9 99614-02-5.rn.

10 bryterol.mp.

11 cedantron.mp.

12 ceramos.mp.

13 emeset.mp.

14 modifical.mp.

15 narfoz.mp.

16 onsia.mp.

17 sakisozin.mp.

18 vomceran.mp.

19 zofrene.mp.

20 zefron.mp.

21 zophron.mp.

22 zophran.mp.

23 zuplenz.mp.

24 zophren.mp.

25 zudan.mp.

26 Granisetron/

27 granisetron$.mp.

28 kytril.mp.

29 BRL-43694.mp.

30 BRL43694.mp.

31 109889-09-0.rn.

32 apf 530.mp.

33 eutrom.mp.

34 granicip.mp.

35 granisol.mp.

36 kevatril.mp.

37 sancuso.mp.

38 taraz.mp.

39 dolasetron.mp.

40 anzemet.mp.

41 anemet.mp.

42 zamanon.mp.

43 MDL 73,147EF.mp.

44 MDL-73147EF.mp.

45 dolasetron.rn.

46 palonosetron.mp.

47 135729-61-2.rn.

48 onicit.mp.

49 aloxi.mp.

50 2-Qhbiqo.mp.

51 Serotonin 5-HT3 Receptor Antagonists/

52 5ht3.mp.

53 5-HT3.mp.

54 "5-Hydroxytryptamine-3 receptor antagonist?".mp.

55 "serotonin type 3 receptor antagonist$".mp.

56 "5-hydroxytryptamine-3 antagonist$".mp.

57 or/1-56

58 chemotherap$.mp.

59 chemo-therap$.mp.

60 Antineoplastic Combined Chemotherapy Protocols/

61 exp Antineoplastic Agents/

62 canc$.mp.

63 carcinoma$.mp.

64 tumo?r$.mp.

65 neoplasm$.mp.

66 on?olog$.mp.

67 surger$.mp.

68 surgical$.mp.

69 su.fs.

70 exp Surgical Procedures, Operative/

71 Nausea/

72 Vomiting/

73 nause$.mp.

74 vomit$.mp.

75 emesis.mp.

76 PONV.mp.

77 "Postoperative Nausea and Vomiting"/

78 or/58-77

79 57 and 78

80 exp Animals/ not (exp Animals/ and Humans/)

81 79 not 80
